# Nanosomes in Precision Nanomedicine (Second Edition)

**DOI:** 10.3390/nano16150952

**Published:** 2026-08-03

**Authors:** Lucia Baldino

**Affiliations:** Department of Industrial Engineering, University of Salerno, via Giovanni Paolo II 132, 84084 Fisciano, Italy; lbaldino@unisa.it

Nanosomes are small vesicles that are used in precision nanomedicine to deliver therapeutic drugs to specific cells or tissues. They are designed to improve the efficacy and safety of drug delivery systems. Nanosomes have a unique structure consisting of a lipophilic bi- or multi-layer around a hydrophilic core. The core contains either a therapeutic drug or a functional biomolecule that can selectively target specific cells or tissues. They can also encapsulate inorganic nanoparticles for anticancer treatments. These nanovesicles can pass through biological barriers, such as the blood–brain barrier, and target specific cells or tissues, thus reducing the side effects associated with traditional drug delivery systems. Thanks to continuous research and development, nanosomes represent a promise in revolutionizing the way to treat diseases, improving patients’ lives through precision nanomedicine.

The Special Issue “Nanosomes in Precision Nanomedicine (Second Edition)” covers this valuable research topic; but it is not limited to this. Indeed, it also includes the latest advances in nanomedicine using other types of biocompatible organic and inorganic nanomaterials.

In this context, Katrivas et al. [1] studied DNA–nanoparticle interactions; in particular, the formation of gold nanoparticles (AuNPs)-DNA nanostructures, due to the strong affinity of nucleobases for the gold surface, to be applied in DNA-based nanoelectronics, biosensing, and self-assembled nanomaterials.

Buonerba et al. [2] produced a nanodevice based on gold nanoparticles (AuNPs) capped with poly(diethylvinylphosphonate) (PDEVP), characterized by photophysical and thermoresponsive properties. This kind of nanodevice can be used in the photoablation of malignant cells or in controlled drug delivery induced by light or heat.

Yun and Kim [3] highlighted the potential of *Scutellaria baicalensis* extract-induced exosomes (SBEIE) in addressing the negative impacts of fine dust exposure on oral health. In particular, compared to SBE, the preventive effectiveness of SBEIEs against fine dust was approximately two times higher. However, further research is required to determine the optimal concentration and to investigate potential side effects through animal or clinical trials.

Zawidlak-Węgrzyńska and Rydz [4] reviewed the recent progress in the design, functionalization, and clinical translation of polymer-based nanoparticles, with a focus on drug delivery, diagnostics, theranostics, nanosponges, and regenerative medicine. This study underlined that challenges remain, including scalable and reproducible production processes, regulatory harmonization, and comprehensive long-term biosafety assessment.

Christoforou et al. [5] investigated the effect of polyethylene glycol (PEG) physicochemical properties on drug delivery, focusing on solid dosage forms and nanovesicles, along with an evaluation of its contribution to the fabrication of safe delivery and theragnostic systems. Indeed, it is a versatile hydrophilic polymer that exhibits high potential in the development of drug delivery systems, either as a coating agent of nanocarriers or in solid dosage form applications.

This overview demonstrates that nanotechnology has a key role in nanomedicine, and nanomaterials, such as nanosomes that may be loaded with organic/inorganic nanoparticles, represent a valid strategy to address the current challenges in targeted and precise drug release. The research is developing in different directions with the common aim of reducing side effects and obtaining a controlled drug release. However, further work is required to develop reproducible and industrially attractive production processes in order to translate the lab-scale research in to effective health-care platforms.

## References

[1] Katrivas L., Proshkina G.M., Deyev S.M., Kotlyar A.B. (2025). DNA Unwinding Driven by Gold Nanoparticles. Nanomaterials.

[2] Buonerba A., Lapenta R., Della Monica F., Piacentini R., Baldino L., Scognamiglio M.R., Speranza V., Milione S., Capacchione C., Rieger B. (2024). Thermo- and Photoresponsive Smart Nanomaterial Based on Poly(diethyl vinyl phosphonate)-Capped Gold Nanoparticles. Nanomaterials.

[3] Yun M., Kim B. (2024). Effects of Scutellaria baicalensis Extract-Induced Exosomes on the Periodontal Stem Cells and Immune Cells under Fine Dust. Nanomaterials.

[4] Zawidlak-Węgrzyńska B., Rydz J. (2026). Polymer Nanoparticles in Medical Applications—Future Directions. Nanomaterials.

[5] Christoforou I., Kalatzis A., Siamidi A., Vlachou M., Pispas S., Pippa N. (2025). The Ubiquitous Use of Polyethylene Glycol in Pharmaceutical Design and Development: Technological Aspects and Future Perspectives. Nanomaterials.

